# Using electronic biology based platform to predict flu vaccine efficacy for 2018/2019

**DOI:** 10.12688/f1000research.14140.2

**Published:** 2018-05-29

**Authors:** Slobodan Paessler, Veljko Veljkovic

**Affiliations:** 1 Department of Pathology, Galveston National Laboratory, University of Texas Medical Branch at Galveston, Galveston , TX, USA; 2 Galveston National Laboratory, Institute for Human Infections and Immunity, University of Texas Medical Branch at Galveston, Galveston, TX, USA; 3 Biomed Protection, Galveston, TX, USA

**Keywords:** influenza, vaccine efficacy, H3N2, electronic biology

## Abstract

Flu epidemics and potential pandemics pose great challenges to public health institutions, scientists and vaccine producers. Creating right vaccine composition for different parts of the world is not trivial and has been historically very problematic. This often resulted in decrease in vaccinations and reduced trust in public health officials. To improve future protection of population against flu we urgently need new methods for vaccine efficacy prediction and vaccine virus selection.

## Introduction

Vaccine effectiveness (VE) against H3N2 viruses is typically lower than VE against influenza H1N1 and/or influenza B viruses. It’s not uncommon to see VE of about 30 percent against H3N2 viruses. Furthermore, during the flu season 2017 in Australia, VE of the seasonal flu vaccine was around 10% resulting in record-high numbers of laboratory-confirmed influenza A infections, hospitalizations and deaths ^1^. This situation raised concerns that similar could happen in the United States during the flu season 2017/2018, in which H3N2 viruses were predominant. The concerns were based on assumptions that H3N2 viruses in Australia and US were similar, as the classical phylogeny indicated, and because the vaccine composition was identical one could expect comparable levels of VE. Therefore, predicted VE of the flu vaccine in the USA at the beginning of the flu season was around 10% ^2^. This prediction was justified and rationalized using the assumption that H3N2 viruses circulating in Australia in the flu season 2016–17 are similar to viruses in the Northern Hemisphere.

Comparison of Australian H3N2 viruses and viruses isolated in the USA at the beginning of the flu season 2017–18, performed using a novel functional phylogenetic tool, demonstrated significant difference between these two groups of viruses ^3^. This new information led us to predict that the flu vaccine in US should work in the season 2017–18 just as well as in 2016–17 ^3^. Our prediction was recently confirmed in the interim CDC estimation of 2017–18 seasonal influenza VE, published and released in February 2018 ^1^. Moreover, the risk for a (H3N2) associated medically-attended influenza illness was reduced through vaccination by 59% among children aged 6 months through 8 years ^1^.

## Methods

To improve VE for the flu season 2018, WHO selected in September 2017 the new vaccine virus A/Singapore/INFIMH-16-0019/2016, which is better adapted to H3N2 viruses circulating in the South Hemisphere ( See WHO recommendation of vaccine compositions for the Southern Hemisphere). The WHO in February 2018 selected the same virus for the vaccine for the season 2018–19 in the North Hemisphere ( See WHO recommendation of vaccine compositions for the Northern Hemisphere).

In order to assess VE against H3N2 viruses for the next flu season 2018–19 in the United States, we analyzed compatibility between new vaccine virus A/Singapore/INFIMH-16-0019/2016 and H3N2 viruses isolated in 2018 in US. This analysis was performed using the informational spectrum method (ISM) based phylogenetic algorithm, the Informational Spectrum-based Phylogenetic Analysis (ISTREE), which we previously used to assess VE for the flu season 2017–18 ^3^. This algorithm, which is based on the informational hallmark of proteins that determines their biological function, was previously described in more detail ^4^.

## Results and discussion

**Figure 1.**
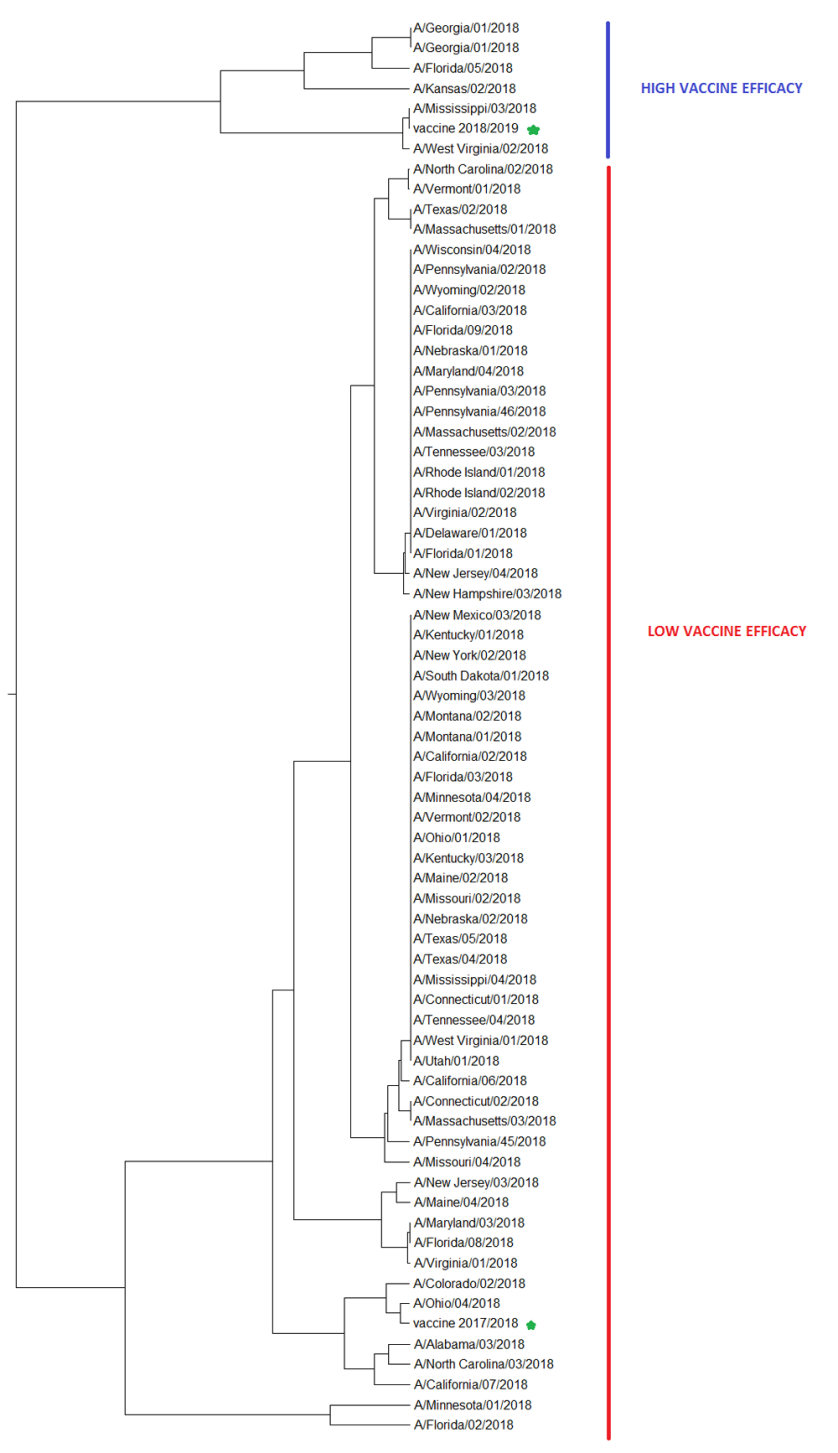
The ISM-based phylogenetic tree of HA1 from human H3N2 influenza viruses collected in the United States from January to February 2018. The vaccine viruses are marked with asterisk (green).

In Figure 1 the ISM-based phylogenetic tree is presented for hemagglutinin HA1 from 68 H3N2 viruses collected in the United States from January to February 2018 and stored in the publicly open database GISAID. As can be seen in this figure, the H3N2 viruses are grouped into two separate clusters and the novel vaccine virus A/Singapore/INFIMH-16-0019/2016 belongs to the small cluster encompassing only 8.8% of analyzed viruses. Previously we showed that 71% of H3N2 viruses isolated in the beginning of the US season 2017–18 were informationally compatible with vaccine virus ^3^. This compatibility resulted in good protection against H3N2 viruses in this season ^1^. The low informational compatibility between new vaccine virus and H3N2 viruses circulating in US suggests that VE for the next flu season in US could be very low. Of note is that H3N2 virus A/Hong Kong/4801/2014 in vaccine for the season 2017–2018 better fits US viruses than new vaccine virus A/Singapore/INFIMH-16-0019/2016. This suggests possibility that VE of the current vaccine could be even higher than that for the new vaccine.

**Figure 2.**
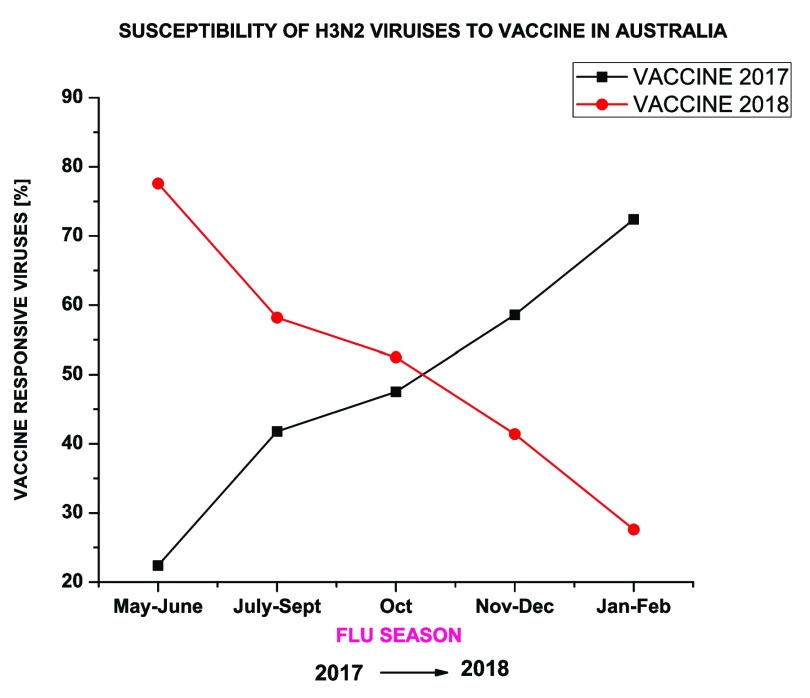
Assessment of the responsiveness to flu vaccines A/Hong Kong/4801/2014 and A/Singapore/INFIMH-16-0019/2016 of H3N2 viruses collected in Australia between May 2017 and February 2018.

Recently, GISAID released data for H3N2 viruses isolated in Australia in January and February, representing precursors of seasonal flu viruses in Australia in 2018 ( Dataset 2. Comparison of these viruses with these collected during the flu season 2017 ( Dataset 3) served as a base for prediction of VE during the next flu season in Australia. Unexpectedly, the results of this analysis showed that the predicted responsiveness to new vaccine A/Singapore/INFIMH-16-0019/2016 continuously decreases from May 2017 to February 2018 and increases for the previous vaccine A/Hong Kong/4801/2014 ( Figure 2). This result suggests the low efficacy of the new vaccine against H3N2 viruses in the next flu season in Australia. Monitoring in next moths of H3N2 viruses in Australia will be necessary for confirmation of this prediction.

We propose the “ISM-based phylogenetic algorithm ISTREE analysis” for rapid and accurate analysis of different influenza A viruses that can be used for VE prediction. This is a first report VE prediction prior to flu season using computational analysis. Our prediction has been recently confirmed through laboratory reports released by CDC. Based on current data, we predict low VE for the season 2018/2019 for Australia and US due to vaccine virus selection.

Human H3N2 influenza viruses collected in the United States from January to February 2018 (GISAID EpiFlu ^TM^ database, accessed February 20, 2018) Click here for additional data file. Copyright: © 2018 Paessler S and Veljkovic V 2018 Data associated with the article are available under the terms of the Creative Commons Zero "No rights reserved" data waiver (CC0 1.0 Public domain dedication).

Human H3N2 influenza viruses collected in Australia from January to February 2018 (GISAID EpiFlu ^TM^database, accessed May 22, 2018) Click here for additional data file. Copyright: © 2018 Paessler S and Veljkovic V 2018 Data associated with the article are available under the terms of the Creative Commons Zero "No rights reserved" data waiver (CC0 1.0 Public domain dedication).

Human H3N2 influenza viruses collected in Australia from May 2017 to December 20178 (GISAID EpiFluTMdatabase, accessed May 22, 2018) Click here for additional data file. Copyright: © 2018 Paessler S and Veljkovic V 2018 Data associated with the article are available under the terms of the Creative Commons Zero "No rights reserved" data waiver (CC0 1.0 Public domain dedication).

## Data availability

The data referenced by this article are under copyright with the following copyright statement: Copyright: © 2018 Paessler S and Veljkovic V

Data associated with the article are available under the terms of the Creative Commons Zero "No rights reserved" data waiver (CC0 1.0 Public domain dedication). 

Sequence data of the viruses were obtained from the GISAID EpiFlu ^TM^ Database. To access the database each individual user should complete the “Registration Form For Individual Users”. This form, together with detailed instructions, are available on the website. After submission of the Registration form, the user will receive a password. There are no any other restrictions for the access to GISAID. Conditions of access to, and use of, the GISAID EpiFlu ^TM^ Database and Data are defined by the Terms of Use.

**Dataset 1:** Human H3N2 influenza viruses collected in the United States from January to February 2018 (GISAID EpiFlu ^TM^ database, accessed February 20, 2018). 10.5256/f1000research.14140.d196223 ^5^

**Dataset 2:** Human H3N2 influenza viruses collected in Australia from January to February 2018 (GISAID EpiFlu ^TM^database, accessed May 22, 2018). 10.5256/f1000research.14140.d204886 ^6^

**Dataset 3:** Human H3N2 influenza viruses collected in Australia from May 2017 to December 20178 (GISAID EpiFlu ^TM^database, accessed May 22, 2018). 10.5256/f1000research.14140.d204887 ^7^
