# Supplementary material for: Using electronic biology based platform to predict flu vaccine efficacy for 2018/2019
Source: F1000Res. 2018 May 29;7:298. Originally published 2018 Mar 8. [Version 2] doi: 10.12688/f1000research.14140.2 (PMC5865198; doi:10.12688/f1000research.14140.2)
Supplement: Peer review history

# Peer review: Referee response for version 2

**DOI:** 10.5256/f1000research.16453.r34493

**Published:** 2018-05-30

**Authors:** Timm C. Harder

**Affiliations:** 1 Federal Research Institute for Animal Health, Friedrich Loeffler Institute, Greifswald, Germany

In their response the authors obviously accepted the necessity of an independent backup of their data. They even mentioned published work that corroborates their previous predictions. It would be much appreciated if they include this link to CDC data in their paper.

I have read this submission. I believe that I have an appropriate level of expertise to confirm that it is of an acceptable scientific standard, however I have significant reservations, as outlined above.

# Peer review: Referee response for version 1

**DOI:** 10.5256/f1000research.15380.r31708

**Published:** 2018-03-27

**Authors:** Timm C. Harder

**Affiliations:** 1 Federal Research Institute for Animal Health, Friedrich Loeffler Institute, Greifswald, Germany

The authors provide an analysis of HA sequences of recent H3N2 influenza viruses from the ongoing season using the protein sequence-based clustering algorithm ISTREE. This method has been developed and published by one of the authors several years ago. ISTREE seeks to combine molecular data and provide a clustering with respect to antigenic relatedness of the influenza virus HA. Previous publications provided a retrospective analysis of genetic and antigenic data. Basically, ISTREE is advertised as an alternative to antigenic cartography of influenza viruses using serologic data based on hemagglutination inhibition (HI).

 In fact, such algorithms would be of great interest and value in terms of vaccine selection. The prospective statements re appropriate vaccine strains for a future influenza season made by the authors here are quite clear but they are solely based on ISTREE assessments. No flanking/supporting data based on standard HI assays is provided.  Assessing the validity of the ISTREE data therefore is not possible. The data may well turn out to be useful but, as it currently stands, they may be totally misleading as well.

 Solid antigenic data will be required to validate the potentially useful ISTREE algorithm.

I have read this submission. I believe that I have an appropriate level of expertise to state that I do not consider it to be of an acceptable scientific standard, for reasons outlined above.

# Response

**Published:** 2018-04-04

**Authors:** Veljko Veljkovic

**Affiliations:** Biomed Protection, Galveston, USA, USA

The major Referee’s criticism concerning our study is the lack of serology/virology data to support our approach for making valid predictions. This criticism misses the major point of the study, which is to rapidly release predictions based on in silico screening to allow other scientists to look at it as early as possible and to test it through the flu season. However, as we have mentioned in our paper we used this method previously and if the reviewer would have read the paper carefully, he would have realized that our prediction for the flu season 2017/18 was confirmed several months later by CDC using various serological assays. These data can be found in CDC laboratory reports (see the latest CDC report: https://www.cdc.gov/flu/weekly/index.htm) and present a totally independent validation of our approach.

# Peer review: Referee response for version 1

**DOI:** 10.5256/f1000research.15380.r31709

**Published:** 2018-03-22

**Authors:** Abdel-Satar Arafa

**Affiliations:** 1 National Laboratory for Veterinary Quality Control on Poultry Production, Animal Health Research Institute, Giza, Egypt

The article “Using electronic biology based platform to predict flu vaccine efficacy for 2018/2019” is clearly presented and technically sound enough for publication in F1000Research online. It is well-written and supported by computational well-developed bioinformatics analysis.

 I just suggest one observations that should be clarified to enhance the work:

 -          Fig 1 The ISM tree based analysis is not supported by a tool to predict grouping like bootstrapping method to confirm accurate grouping

 Finally,  the article is recommended for publication in its present format.

I have read this submission. I believe that I have an appropriate level of expertise to confirm that it is of an acceptable scientific standard.

# Peer review: Referee response for version 1

**DOI:** 10.5256/f1000research.15380.r31711

**Published:** 2018-03-22

**Authors:** Daniel R. Perez

**Affiliations:** 1 Department of Population Health, Poultry Diagnostic and Research Center (PDRC), University of Georgia, Athens, GA, USA

This is a provocative research article based on the authors' previous development of the informational spectrum method that takes into account how virus/receptor interactions modulate the antigenic cross-reactive phenotype of the HA protein of influenza A viruses. Using this method, the authors were able to shed light on the lack of efficacy of the vaccine against the H3 subtype during the 2014/15. They were also able to predict that in the US, the vaccine against the H3 subtype was going to be more efficacious than previously anticipated by the CDC. Later in the 2017/18 season, the CDC confirmed the authors' prediction. In this latest article, the authors predict that the selection of the vaccine candidate for the 2018/19 is less than ideal and suggest that vaccine efficacy against the H3 subtype will be lower than in this past season. This type of work is fascinating and necessary and hopefully it becomes part of the toolkit for the selection of vaccine candidates against influenza.

I have read this submission. I believe that I have an appropriate level of expertise to confirm that it is of an acceptable scientific standard.
